# Involvement of GPR50 polymorphisms in depression: independent replication in a prospective elderly cohort

**DOI:** 10.1002/brb3.313

**Published:** 2015-02-08

**Authors:** Joanne Ryan, Isabelle Carrière, Karen Ritchie, Marie-Laure Ancelin

**Affiliations:** 1 Inserm, U1061Montpellier, F-34093, France; 2 Univ Montpellier 1, U1061Montpellier, France; 3 CDE, Murdoch Childrens Research Institute, Royal Children's HospitalParkville, Victoria, 3052, Australia; 4 Department of Paediatrics, University of MelbourneParkville, Victoria, 3052, Australia; 5 Faculty of Medicine, Imperial CollegeLondon, W12 0NN, U.K

**Keywords:** Antidepressants, candidate gene, GPCR, *GPR50*, late-life depression, lipid levels

## Abstract

**Introduction:**

Despite the explosion in genetic association studies over the last decade, clearly identified genetic risk factors for depression remain scarce and replication studies are becoming increasingly important. G-protein-coupled receptor 50 (*GPR50*) has been implicated in psychiatric disorders in a small number of studies, although not consistently.

**Methods:**

Data were obtained from 1010 elderly men and women from the prospective population-based ESPRIT study. Logistic regression and survival models were used to determine whether three common *GPR50* polymorphisms were associated with depression prevalence or the incidence of depression over 12-years. The analyses were adjusted for a range of covariates such as comorbidity and cholesterol levels, to determine independent associations.

**Results:**

All three variants showed some evidence of an association with late-life depression in women, although these were not consistent across outcomes, the overall effect sizes were relatively small, and most would not remain significant after correction for multiple testing. Women heterozygous for *rs13440581,* had a 1.6-fold increased risk of baseline depression, while the odds of depression comorbid with anxiety were increased fourfold for women homozygous for the minor allele of *rs2072621*. When depressed women at baseline were excluded from the analysis, however, neither variant was associated with the 12-year incidence of depression. In contrast, *rs561077* was associated with a 1.8-fold increased risk of incident depression specifically. No significant associations were observed in men.

**Discussion:**

Our results thus provide only weak support for the involvement of *GPR50* variants in late-life depression, which appear specific to certain subgroups of depressed individuals (i.e., women and those with more severe forms of depression).

## Introduction

G-protein-coupled receptors (GPCRs) are a superfamily of protein receptors which play a key role in cell signaling and have a wide range of physiological functions. These seven-transmembrane domain receptors undergo a conformational change upon ligand binding, which in turn triggers signal transduction inside the cell and regulates a cascade of downstream processes. GPCRs are thus currently considered as key targets for drug development (Violin et al. 2014).

The melatonin-related receptor or GPCR 50 (*GPR50*) encodes an integral membrane protein located on the X-chromosome (Xq28) and found exclusively in mammals (Li et al. 2013). This receptor is closely related to melatonin receptors 1 and 2, sharing 45% sequence homology (Reppert et al. 1996), and can bind both subtypes forming heterodimers. *Gpr50,* however, cannot bind melatonin and remains an orphan receptor with no known ligands. While the exact function of this receptor thus remains unclear, it can disrupt melatonin signaling through binding to melatonin receptor 1 (Levoye et al. 2006) and appears to play a role in lipid metabolism (Bhattacharyya et al. 2006) and energy homeostasis (Ivanova et al. 2008). Furthermore, *GPR50* has been shown to interact with genes involved in neural development (Grunewald et al. 2009) and its high levels of expression in the pituitary and hypothalamus (Reppert et al. 1996; Vassilatis et al. 2003; Sidibe et al. 2010), indicate it could play a role in neurotransmitter signaling and the stress response. Indeed, Gpr50 has been shown to modulate activity of the transcriptional coactivator TIP60, which in turn influences glucocorticoid receptor signaling (Li et al. 2011). This suggests that *GPR50* could be involved in the vulnerability to neuropsychiatric disorder, especially in the elderly who constitute a particularly important group, having accumulated stressful events across the life-time, with unique patterns of stress response and affective disorder characteristics.

Findings from association studies suggest that it may be a candidate gene for affective disorders, including depression. A Scottish case–control study of hospital patients reported female-specific associations between *GPR50* variants and three psychiatric conditions (bipolar disorder, major depressive disorder [MDD], schizophrenia) (Thomson et al. 2005). However, subsequent studies in children and adults have reported mixed findings (Alaerts et al. 2006; Feng et al. 2007; Macintyre et al. 2010). Thus, the exact role of *GPR50* remains unclear and further investigation in other populations, including the elderly, is warranted, especially given the relatively high heritability of depression but lack of clearly identified genetic risk factors to date. None of the prior studies investigating *GPR50* have considered antidepressant treatment or anxiety comorbidity either, which is frequent in the elderly (Beekman et al. 2000) and may reflect greater severity of the disorder (Lenze 2003). Furthermore, no prior study has considered confounding factors in their analysis which could influence the associations. Importantly, as *GPR50* variants appear to influence circulating lipid levels (Bhattacharyya et al. 2006) and disrupted lipid metabolism is often seen in patients with affective disorders and more specifically depression (Sagud et al. 2009), analysis controlling for such measures is thus clearly needed.

This study aims to investigate the role of *GPR50* gene variants in late-life depression, taking into account antidepressant use and comorbid anxiety, which is frequent in the elderly, while controlling for lipid levels.

## Materials and Methods

### Participants

Data used for this study came from the prospective ESPRIT study of neuropsychiatric disorders in French elderly, with random recruitment of men and women living in the Montpellier region and aged at least 65 years (Ritchie et al. 2004). The study protocol was approved by the Ethical Committee of the University Hospital of Kremlin-Bicêtre and all procedures were undertaken with the adequate understanding and written consent of the participants. Interviews and clinical examinations were administered by trained staff at baseline and after 2-, 4-, 7-, 10-, and 12-years of follow-up.

Of the 2199 nondemented participants recruited to the study, 1076 provided buccal samples for genotyping analysis, and 94.9% had complete and validated genotyping data for all single nucleotide polymorphisms (SNPs). Other participants were excluded from the current analysis because they were not assessed for depression at baseline (*n* = 5), or were missing data concerning key covariates (*n* = 6), leaving a sample of 1010 for the analysis of prevalent depression at baseline. When 12-year depression incidence was examined, those with baseline depression were excluded from the analysis. Participants included in this analysis were less likely to be depressed, use antidepressants and had fewer incapacities, cognitive impairment and cardiovascular disease (*P* < 0.001) but were more likely to live with others and be better educated. Males included in this analysis were also less likely to carry the G allele of *rs561077* (*P* = 0.03).

### Depression and anxiety measures

All participants in the study were drawn from the general population and were assessed for psychiatric disorders at baseline and at every follow-up. A standardized psychiatric examination, the Mini-International Neuropsychiatry Interview (MINI), was used to investigate lifetime and current psychiatric disorder (Sheehan et al. 1998). The MINI provides an extensive symptomatological examination according to the Diagnostic and Statistical Manual of Mental Disorders (DSM-IV) criteria, with diagnostic algorithms applied to assess ‘caseness’, and positive cases were further reviewed by a panel of psychiatrists to validate the initial diagnosis (Ritchie et al. 2004). This examination is commonly used in both clinical and research settings, and has been validated for use in the general population. A hierarchical approach is not used with the MINI, and under each disorder, comorbidity is recorded. DSM-IV Axis 1 psychiatric disorders investigated in this study include current and past MDD, as well as anxiety disorders (all types of phobia, generalized anxiety disorder, obsessive-compulsive disorder, and panic disorder). Depression and anxiety disorders were the most frequently reported psychiatric disorders in this population. Information was also available on the frequency of episodes and age at which the first episode occurred, however, this information was not considered here, to avoid further subgrouping for the analysis.

The presence of current depressive symptoms was assessed using the Center for Epidemiologic Studies Depression Scale (CES-D) (Radloff 1977), which has been validated for use in the elderly, with good sensitivity and specificity. This is a 20-item questionnaire that asks participants to rate their feelings and experiences over the last week, with questions asking specifically about depression-related symptoms, such as feelings of loneliness, loss of appetite, sleep problems, and general mood, and how frequently these occurred on a scale of 0 to 3 for each item (with 0 corresponding to never or very rarely and three indicating most or all the time). From a maximum score of 60, a cutoff of 16 or more on the CES-D is indicative of high levels of depressive symptomatology (Radloff and Locke 1986). A clinically significant level of depression was defined as a current diagnosis of MDD or those scoring above the cutoff on the CES-D (as described previously (Ancelin et al. 2013)). Current use of antidepressants was validated by presentation of the prescription or the medication itself.

### Genotyping

DNA was extracted from buccal samples, as described previously (Freeman et al. 2003; Ancelin et al. 2013) and *GPR50* genotyping was performed by LGC Genomics (Hoddesdon, UK) using a competitive allele-specific PCR incorporating a FRET quencher cassette (http://www.lgcgroup.com/services/genotyping/#.VGQTgWfHSCg). This KASP SNP genotyping system has an error rate of <1%. Fluorescence scanning in a BMG labtech Pherastar scanner was used for the analysis of amplified PCR products and the KlusterCaller 1.1 software enabled interpretation of the results.

The *GPR50* gene is 1854 bp long and is located on the X-chromosome (Xq28). It encodes a 618 amino acids protein with seven transmembrane hydrophobic segments and a long C-terminal tail (Li et al. 2013). As our study aimed to replicate and further clarify previous findings, three SNPs were chosen for analysis based on their reported association with psychiatric disorders, while having potential functional significance. This included two common coding polymorphisms SNPs located in exon 2 of the gene, *rs13440581* and *rs561077*. These nonsynonymous SNPs are often referred to in the literature as Val^606^Ile and Thr^532^Ala, respectively, which indicates the resulting change in amino acid sequence and its position relative to the full-length protein. *Rs561077* is in complete linkage disequilibrium with a 12-bp insertion/deletion polymorphism in the same gene region (sometimes designated as Δ502-505) (Thomson et al. 2005), and as such, Δ502–505 was not genotyped. The third polymorphism genotyped was *rs2072621*, an intronic SNP located between exons 1 and 2 which could still influence gene splicing (Zhou et al. 2014) and has been associated with seasonal affective disorder in females (Delavest et al. 2012). There is relatively weak linkage disequilibrium between all SNPs, as indicated previously (Thomson et al. 2005) the strongest being between rs*561077 and rs13440581* (*D*’ = 0.50 and *r*^*2*^ = 0.24 in females<0.10), whereas the remaining pairwise combination were all <0.10 for both *D*’ and *r*^*2*^).

### Other measures

Detailed information was gathered on the participants socio-demographic status, lifestyle, and behaviors, as well as health-related information. Body mass index (BMI) was calculated as weight (kg) divided by the height squared (m^2^). Activity limitations were assessed with the Rosow and Breslau mobility scale (Rosow and Breslau 1966) and the Instrumental Activities of Daily Living (IADL) scales (Lawton and Brody 1969), to determine the capacity of the individual to undertake common daily tasks unassisted. More specifically, the Rosow and Breslau scale assess mobility, the ability to do heavy housework, walk half a mile, and climb stairs, whereas the IADL is concerned with common activities such as using the telephone, cooking and keeping a budget. The presence of limitations been defined as the inability to complete one or more activities from both scales. Global cognitive function was assessed with the Mini-Mental State Examination (MMSE), as impairment was defined as having a score <24 (Folstein et al. 1975). All participants were examined by a neurologist and a standard clinical protocol was used for the diagnoses and classification of dementia based on DSM-IV criteria. A panel of expert neurologists reviewed all incident cases of dementia independently from study investigators (Ryan et al. 2014). As mentioned above, only nondemented participants were included in this analysis.

Information on the participant's health was obtained through detailed medical questionnaires, a complete inventory of all drugs used within the preceding month and from 12-h fasting blood samples. These questionnaires included their history of cardiovascular diseases (angina pectoris, myocardial infarction, stroke, cardiovascular surgery, or arteritis), or other chronic illnesses such as diabetes (fasting glucose ≥7.0 mmol/L or reported treatment), and hypertension (resting blood pressure ≥160/95 mm Hg or treated). Circulating lipid levels (high- and low-density lipoproteins, HDL and LDL, respectively; total cholesterol and triglycerides) were measured in serum using routine enzymatic methods. LDL cholesterol was determined by the Friedwald formula (Dupuy et al. 2008). Hypercholesterolemia was defined as total cholesterol ≥6.2 mmol/L or reported treatment.

### Statistical analysis

The *GPR50* gene is X-linked so all analyses were undertaken separately in males and females. Hardy–Weinberg equilibrium (HWE) was examined by comparing the observed and expected genotype frequencies in women. As we had no strong a priori evidence for the underlying genetic model (i.e., how the risk for heterozygotes would compare with that of the two homozygotes), association tests in women were performed using a general genetic model which retained the three distinct genotype classes (Lunetta 2008). Linkage disequilibrium between the SNPs was calculated using Haploview version 4.2 (Barrett et al. 2005). The association between baseline socio-demographic and clinical variables with both depression and *GPR50* polymorphisms was examined using *t*-tests, ANOVA and chi-squared tests. Chi-squared analysis was used to determine the association between *GPR50* polymorphisms and prevalent depression at baseline, as well as antidepressant use. Multivariate logistic regression models were then constructed, adjusting for covariates which were found to be associated with depression in this population using the higher cutoff of *P* > 0.10, as well as depression and anxiety comorbidity. Adjustment for lipid levels and cardiovascular disease in the final multivariate models was also examined, as were interaction terms between these factors and gene variants on the association with depression, given previous findings in the literature.

For study participants without depression at baseline, Cox proportional hazards models with delayed entry were used to assess the association between *GPR50* polymorphisms and the 12-year incidence of depression. To avoid the problem of nonproportionality in depression risk with age, age was taken as the basic time scale and birth as the time origin (Thiebaut and Benichou 2004). Multivariate analyses adjusted for the same covariates that were identified previously. There was no indication of colinearity between the covariates in the adjusted models. SAS version 9.4 (SAS Institute, Inc., Cary, NC) was used for all the analyses and all tests were two-tailed. Given that three SNPs were examined separately in males and females, the Bonferroni corrected significance level was *P* < 0.0083.

## Results

The study was comprised of 400 men and 610 women, all aged between 65 and 88 years (Table 1). At baseline, 25.7% of the participants were identified as having clinically significant depression, which included 2.1% with a current MDD, and the remaining with high levels of depressive symptomatology (CES-D ≥ 16). Antidepressants were used by 3.9% of the population. The prevalence of current anxiety disorders was 16.6%, of which 6.8% of all participants had anxiety comorbid with depression. Women were significantly more likely to have current depression or anxiety than men, and were also more likely to use antidepressants (*P* < 0.001 for all comparisons).

**Table 1:** Participant's characteristics at inclusion (*N* = 1010)

Characteristic	Men (*n* = 400)	Women (*n* = 610)
	Median (IQR)	
Age (years)	71 (68–74)	71 (68–75)
Body Mass Index, BMI (kg/m^2^)	25.5 (23.7–27.4)	23.7 (21.6–26.6)

	%	
≥12 years of schooling	47.3	31.0
Married or living with others	6.0	36.0
Current heavy drinker (≥24 grams each day)	38.5	6.1
Heavy smoker (1 pks/year for 10 years)	67.4	21.5
Physical activity limitations	2.0	2.6
Cardiac ischemic pathologies^1^	16.0	7.5
Diabetes (Fasting glucose ≥7 mmol/L or treated)	11.3	4.0
Hypertension (Resting blood pressure ≥160/95 mmHG or treated)	48.5	41.6
Cognitive impairment (MMSE <24)	3.3	8.4
Current depression (CESD ≥ 16 or current MDD)	16.3	32.0
Current anxiety disorder	9.3	21.5
Antidepressant use	1.3	5.6

	Mean (SD)	
Total cholesterol, mmol/L	5.65 (0.90)	6.04 (0.99)
High-density lipoprotein (HDL) cholesterol, mmol/L	1.42 (0.32)	1.77 (0.38)
Low-density lipoprotein (LDL) cholesterol, mmol/L	3.62 (0.81)	3.74 (0.89)
Triglycerides (log, geometric mean), mmol/L	1.20 (1.55)	1.18 (1.50)

1 A history of stroke, myocardial infarction, angina pectoris, cardiovascular surgery or arteritis.

The frequencies of the *GPR50* genotypes were all in HWE (*P* > 0.05). In chi-squared analysis, associations between *GPR50* SNPs and depression or antidepressant use were identified in women only (Table 2). The *rs13440581* polymorphism was associated with both depression and antidepressant use, while there was weak evidence of an association between *rs561077* and antidepressant use specifically. Considering both outcomes together in women, those who were using antidepressants but still meeting the criteria for depression, were much more likely to be *rs13440581* heterozygotes than the women in the three other categories, and less likely to be homozygotes for the minor allele (*P* = 0.003). Due to the small numbers of women in these groups, however, these associations could not be investigated further.

**Table 2:** Frequency of *GPR50* polymorphisms according to depression prevalence and antidepressant use in men and women (*N* = 1010)

	Depression			Antidepressant use			Depression and/or antidepressant use				
	No % (*N*)	Yes% (*N*)	(*χ*^2^)^1^ *P*	No% (*N*)	Yes% (*N*)	*χ*^2^ (df) *P*	No, No% (*N*)	Yes, No% (*N*)	No, Yes% (*N*)	Yes, Yes% (*N*)	*χ*^2^ (df) *P*
Men											
*rs13440581*: G	62.1 (208)	55.4 (36)	(1.03)	61.3 (242)	40 (2)	(0.94)					
A	37.9 (127)	44.6 (29)	0.31	38.7 (153)	60 (3)	0.33					
*rs2072621*: C	58.2 (195)	61.5 (40)	(0.25)	58.2 (230)	100 (5)	(3.56)					
A	41.8 (140)	38.5 (25)	0.62	41.8 (165)	0 (0)	0.06					
rs561077: A	60.9 (204)	60 (39)	(0.02)	60.8 (240)	60 (3)	(0.001)					
G	39.1 (131)	40 (26)	0.89	39.2 (115)	40 (2)	0.97					
Women											
*rs13440581*: GG	33.0 (137)	27.7 (54)	(9.59)	32.1 (185)	17.7 (6)	(10.3)	33.3 (134)	29.5 (51)	25.0 (3)	13.6 (3)	(12.1)
GA	42.9 (178)	55.9 (109)	0.008	45.6 (262)	73.5 (25)	0.006	42.7 (172)	52.0 (90)	50.0 (6)	86.4 (19)	0.003
AA	24.1 (100)	16.4 (32)		22.4 (129)	8.8 (3)		24.1 (97)	18.5 (32)	25.0 (3)	0 (0)	
*rs561077*: AA	32.8 (136)	34.4 (67)	(3.86)	32.3 (186)	50.0 (17)	(6.35)	33.0 (133)	27.2 (47)	25.0 (3)	27.3 (6)	(2.61)
GA	48.9 (203)	41.5 (81)	0.15	47.7 (275)	26.5 (9)	0.04	46.2 (186)	45.7 (79)	33.3 (4)	59.1 (13)	0.27
GG	18.3 (76)	24.1 (47)		20.0 (115)	23.5 (8)		20.8 (84)	27.1 (47)	41.7 (5)	13.6 (3)	
*rs2072621*: CC	32.8 (136)	27.2 (53)	(2.42)	31.3 (80)	26.5 (9)	(0.36)	32.3 (130)	32.4 (56)	50.0 (6)	50.0 (11)	(5.98)
AC	45.8 (190)	47.2 (92)	0.30	46.0 (265)	50.0 (17)	0.84	49.9 (201)	42.8 (74)	16.7 (2)	31.8 (7)	0.07
AA	21.4 (89)	25.6 (50)		22.7 (131)	23.5 (8)		17.9 (72)	24.9 (43)	33.3 (4)	18.2 (4)	

1 Degrees of freedom = one for men (i.e., two group genotypes); two for women (three group genotypes).

Given that confounders could help account for the observed associations, analyses were then adjusted for covariates found to be significantly associated with depression in this population, and which could themselves be associated with *GPR50* polymorphisms. After adjustment the association between *rs13440581* and depression in women remained significant (*P*-global = 0.004), with heterozygote individuals having a 1.6-fold increased risk of depression compared to homozygotes for the major allele (Table 3). This association decreased in significance when antidepressant use was included in the multivariate models (OR = 1.53, 95% CI = 1.01–2.34, *P* = 0.046), although the odds ratio remained relatively stable. In terms of *rs2072621*, there was some evidence that the presence of a current anxiety disorder modified the association between this SNP and the risk of depression (*P*-interaction = 0.07). Indeed, in stratified analysis, women homozygote for the minor allele of *rs2072621* had a fourfold increased risk of depression in those with anxiety also, whereas in women without anxiety, *rs2072621* was not associated with an increased risk despite a greater number of cases. Of note, all these associations were not influenced by lipid levels, as inclusion of the variables for HDL or LDL cholesterol, total cholesterol, or triglycerides did not modify the associations shown in Table 3. Furthermore, interaction terms between the SNPs and levels or cardiovascular disease were not significant.

**Table 3:** Multivariate adjusted association between *GPR50* polymorphisms and depression prevalence in women (*N* = 610)

	Odds of Depression^1^								
	OR (95% CI)	Chi-squared^2^	*P*						
*rs13440581*: GG	1								
GA	1.64 (1.08–2.50)	5.36	0.02						
AA	0.78 (0.46–1.34)	0.79	0.37						
*rs561077*: AA	1								
GA	0.83 (0.55–1.26)	0.78	0.38						
GG	1.24 (0.72–2.04)	0.74	0.39						

				No current anxiety (*n = 478*)			Current anxiety (*n = 132*)		
				OR (95% CI)	Chi-squared (df)	*P*	OR (95% CI)	Chi-squared (df)	*P*
*rs2072621*: CC	1			1			1		
AC	1.20 (0.79–1.84)	0.72	0.40	1.15 (0.70–1.87)	0.30	0.58	1.43 (0.55–3.71)	0.53	0.47
AA	1.35 (0.81–2.23)	1.35	0.25	0.94 (0.51–1.72)	0.04	0.84	4.05 (1.28–12.9)	5.62	0.02

1 Adjusted for age, education, incapacities, MMSE, cardiovascular ischemic pathologies and current anxiety disorders.

2 One degree of freedom.

The association between *GPR50* polymorphisms and the 12-year incidence of depression in women was then examined, while excluding the 195 women with prevalent depression at baseline (Table 4). An association between *rs561077* and the risk of incident depression (*P*-global =0.009) was found, with homozygotes for the minor allele having 1.8-fold increased risk compared to those homozygous for the major allele. Further adjustment for antidepressant use or anxiety disorder did not influence the associations. No significant association was seen with the heterozygote for *rs13440581,* as observed for prevalent depression, although there was a very weak trend (*P* = 0.07).

**Table 4:** Association^1^ between *GPR50* polymorphisms and the incidence of depression over 12-years in women not depressed at baseline (*n* = 415)

	12-year incident depression				Multivariate adjusted^1^		
	No, *N*	Yes, *N*	Chi-squared^2^	*P*	HR (95% CI	Chi-squared^2^	*P*
*rs13440581*: GG	87	50	2.64	0.27	1		
GA	97	81			1.39 (0.98–1.99)	3.32	0.07
AA	57	43			1.32 (0.87–1.99)	1.70	0.19
*rs2072621*: CC	70	66	3.64	0.16	1		
AC	117	73			0.76 (0.54–1.06)	2.67	0.10
AA	54	35			0.79 (0.52–1.19)	1.26	0.26
*rs561077*: AA	82	54	8.31	0.01	1		
GA	126	77			0.94 (0.66–1.33)	0.12	0.73
GG	33	43			1.77 (1.18–2.67)	7.54	0.006

1 Adjusted for age, education, incapacities, MMSE, and cardiovascular ischemic pathologies.

2 Two degrees of freedom.

## Discussion

Despite the explosion in psychiatric genetics and the exponential increase in the number of gene association studies, very few candidate genes for depression have clearly been identified. The field is plagued by a lack of sufficiently powered replication studies in well-characterized populations. Our prospective study in the general elderly population has attempted to replicate earlier findings of a predominantly female-specific role for *GPR50* in depression, while controlling for potential confounding or mediating factors. All three *GPR50* variants examined showed some evidence of an association in women only, although most associations did not reach Bonferroni corrected significance levels, the overall effect sizes were relatively small and consistent findings were not identified across outcomes. Together this suggests that *GPR50* does not appear to be a good candidate gene for depression.

Linkage studies have implicated the Xq28 gene region in manic depression (Bocchetta et al. 1999) and childhood-onset depressive and bipolar disorders (Wigg et al. 2009). Candidate gene studies have also reported associations between variants in the *GPR50* gene and psychiatric disorders in women. This include associations between bipolar disorder and the deletion Δ502–505 (Thomson et al. 2005), between schizophrenia and *rs2072621* (Thomson et al. 2005) and seasonal affective disorder with *rs2072621* (Delavest et al. 2012), whereas studies of autism spectrum disorder and attention deficient hyperactive disorder have reported null findings (Jonsson et al. 2010; Chaste et al. 2011). The small numbers of studies investigating depression have reported mixed findings. The first case–control study of 226 patients with MDD and 562 ethnically matched controls found that two *GPR50* polymorphisms (*rs13440581* and Δ502–505) were strongly associated with an increased risk of MDD in women (Thomson et al. 2005). On the other hand, another case–control study of 359 MDD patients and 913 controls undertaken in a very similar population (Macintyre et al. 2010), failed to find a significant association with four *GRP50* polymorphisms, including the two identified as significant in the prior study. When they examined more specifically a subset of 56 participants with early onset MDD and current depression, they did report some associations of borderline significance with Δ502–505 in women (4/8 neuropsychological features examined), and with a MDD family history in men. However, a study of childhood-onset mood disorder in 384 families and their children, failed to find any significant associations *rs13440581, rs561077,* and *rs2072621* (Feng et al. 2007). This gene has not been identified in genome-wide association studies, however, there is generally insufficient coverage of SNPs in this region (http://www.hapmap.org).

We investigated two common SNPs, *rs13440581* and *rs561077* (the latter being in complete linkage disequilibrium with Δ502–505), which both result in an amino acid change, thus having potential functional significance. *Rs13440581* was associated with prevalent depression in women, with a similar although non-significant finding for depression incidence. It was also associated with an increased likelihood of using antidepressants and possibly resistant depression with significant symptomatology despite the treatment, although this hypothesis could not be investigated further. *Rs561077* was not associated with depression prevalence, but was associated with a 12-year increased risk of incident depression (*P* = 0.009). These results thus support the earlier work by Thomson (Thomson et al. 2005), although the effect size is quite weak (1.8-fold increased risk). This could be explained by the “winners curse”, where a reduced effect size is commonly observed in replication studies (Ioannidis 2008).

The other SNP examined in our study, *rs2072621*, was associated with a fourfold increased risk of depression comorbid with anxiety, but for women with depression in the absence of anxiety, there was no significant association. High levels of anxiety in depression have been associated with higher suicide risk, longer duration of illness, and greater likelihood of treatment nonresponse (Lenze 2003) and these mixed states may constitute a specific diagnostic entity relatively common among older people (Wolitzky-Taylor et al. 2010; Carter et al. 2012). While this is an intronic nonsynonymous SNP, it could still influence gene splicing and thus has the potential to be a functional variant. It is also possible that this SNP is in linkage disequilibrium with another as yet unidentified variant. A recent study of seasonal affective disorder, also found a weak association with this SNP in females only (Delavest et al. 2012). Yet, prior studies which have focused on MDD failed to find a significant association. No other studies to date have investigated anxiety or depression anxiety comorbidity, and our result thus requires replication to rule out the possibility that it is simply a chance finding, especially given the relatively low number of comorbid cases.

All the prior studies of *GPR50* and depression are based on univariate analysis. While one could argue that confounding is less problematic in genetic association studies, it remains that if *GPR50* variants influence health-related outcomes, this could explain their association with depression (depression itself being associated with worse health). *Rs13440581* and *rs561077* variants have been associated with elevated circulating triglyceride and lower HDL-cholesterol levels, suggesting they help regulate lipid metabolism (Bhattacharyya et al. 2006). Varying lipid levels have also been linked with affective disorders (Sagud et al. 2009) and we have previously reported an association between low HDL cholesterol and depression in elderly women specifically (Ancelin et al. 2010). In our study, there was only a nonsignificant trend for an association between *rs2072621* and lipid levels (HDL-cholesterol *P* = 0.08; trigylcerides *P* = 0.07) and adjustment for any of the lipid measures did not influence the associations found.

The question remains of how could *GPR50* influence an individual's risk of depression and why do the associations appear specific to women. *GPR50* is an X-linked orphan receptor with no known ligands and its function remains unclear. It is expressed early in embryonic development and predominates in brain regions implicated in neurotransmitter signaling (Grunewald et al. 2012) and could play a key role in regulating the Hypothalamic-Pituitary-Adrenal HPA axis. In keeping with this, *GPR50* has been shown to modulate glucocorticoid receptor signaling (Li et al. 2011). Sex-specific associations between glucocorticoid receptor gene variants and HPA axis responses to stress as well as opposite effects of female and male steroids on the HPA axis have previously been reported (Kumsta et al. 2007; Chrousos 2009). This may help explain the gender-specific associations, and disruption of the HPA axis is often seen in people with depression (De Kloet et al. 2005; Beluche et al. 2009). It is also possible that *GPR50's* association with depression occurs via its ability to modulate melatonin signaling, as melatonin reportedly plays a role in depression (Singh and Jadhav 2014). While *GPR50* does not bind melatonin itself, it has been shown to inhibit binding of melatonin to the melatonin receptor 1 (Levoye et al. 2006), and thus influences signaling.

Strengths of our study are that it was population-based and involved more than 1000 elderly who were followed for over 12 years. However, people who agree to participate in studies of this kind are generally of a higher education and in better health than the population as a whole, and we could only include participants who had been assessed for depression, thus reducing the overall power of the study. However, depression was assessed by trained staff using two distinct measures validated in the general population, including a structured diagnostic interview (Radloff 1977; Sheehan et al. 1998) which was verified by a panel of psychiatrists according to DSM-IV criteria. Antidepressant use was validated by presentation of the medication or prescription. Our study is also strengthened by extensive health and lifestyle data on all participants, including measures of circulating lipid levels, which allow adjustment for a wide-range of potential confounders. The genotyping system had a very low error rate, and we were able to control for accuracy through some duplicate samples. Unfortunately, as we could not question participants about their ethnicity due to French law, we were not able to control for this in our analysis. However, prior genotyping data from a subsample of participants indicates that the population is more than 99% Caucasian (unpubl. data and (Lambert et al. 2009)).

## Conclusion

Our prospective study in the general population has helped clarify the role of *GPR50* in mood disorders, by investigating the association between three commonly studied nonsynonymous variants and depression, comorbid depression and anxiety, as well as antidepressant use in the elderly. Our results provide evidence of a weak female-specific association between *GPR50* variants and late-life depression, which could be specific for more severe depression (i.e., depression comorbid with anxiety, as well as depression despite antidepressant treatment). However, given that findings were not consistent across all outcomes, and that the majority of associations would not remain significant after correction for multiple testing, despite our relatively large sample size, this suggests little support for *GPR50* as a good candidate gene for late-life depression.
